# Activin a signaling regulates cell invasion and proliferation in esophageal adenocarcinoma

**DOI:** 10.18632/oncotarget.5349

**Published:** 2015-10-06

**Authors:** Chase Taylor, Holli A. Loomans, Gregoire F. Le Bras, Rainelli B. Koumangoye, Alejandra I. Romero-Morales, Laura L. Quast, Alexander I. Zaika, Wael El-Rifai, Thomas Andl, Claudia D. Andl

**Affiliations:** ^1^ Departments of Surgery, Vanderbilt University Medical Center, Nashville, TN 37232-6840, USA; ^2^ Cancer Biology, Vanderbilt University Medical Center, Nashville, TN 37232-6840, USA; ^3^ Vanderbilt Ingram Cancer Center, Vanderbilt University Medical Center, Nashville, TN 37232-6840, USA; ^4^ Vanderbilt Digestive Disease Center, Vanderbilt University Medical Center, Nashville, TN 37232-6840, USA; ^5^ Division of Dermatology, Department of Medicine, Vanderbilt University Medical Center, Nashville, TN 37232-6840, USA

**Keywords:** barrett's esophagus, esophageal adenocarcinoma, SOX9

## Abstract

TGFβ signaling has been implicated in the metaplasia from squamous epithelia to Barrett's esophagus and, ultimately, esophageal adenocarcinoma. The role of the family member Activin A in Barrett's tumorigenesis is less well established. As tumorigenesis is influenced by factors in the tumor microenvironment, such as fibroblasts and the extracellular matrix, we aimed to determine if epithelial cell-derived Activin affects initiation and progression differently than Activin signaling stimulation from a mimicked stromal source. Using Barrett's esophagus cells, CPB, and the esophageal adenocarcinoma cell lines OE33 and FLO-1, we showed that Activin reduces colony formation only in CPB cells. Epithelial cell overexpression of Activin increased cell migration and invasion in Boyden chamber assays in CPB and FLO-1 cells, which exhibited mesenchymal features such as the expression of the CD44 standard form, vimentin, and MT1-MMP. When grown in organotypic reconstructs, OE33 cells expressed E-cadherin and Keratin 8. As mesenchymal characteristics have been associated with the acquisition of stem cell-like features, we analyzed the expression and localization of SOX9, showing nuclear localization of SOX9 in esophageal CPB and FLO-1 cells.

In conclusion, we show a role for autocrine Activin signaling in the regulation of colony formation, cell migration and invasion in Barrett's tumorigenesis.

## INTRODUCTION

Esophageal adenocarcinoma (EAC) is often thought to arise from a clonal stem-like population of cells, which is potentially responsible for its poor prognosis. Transforming growth factor β (TGFβ) and Notch signaling pathways play important roles in regulating self-renewal of stem cells and cell-fate determination. Both pathways are frequently implicated in Barrett's tumorigenesis [1]. It has been shown that loss of members of the TGFβ signaling cascade, such as Smad4 and β2 spectrin, can contribute to the initiation of Barrett's esophagus and the progression to esophageal adenocarcinoma through concomitant upregulation of Notch targets Hes1 and Jagged1 [2]. Similarly, analysis of a panel of esophageal adenocarcinoma cell lines demonstrated failed cell cycle arrest after TGFβ stimulation, as they did not respond with the expected down-regulation of c-Myc or the induction p21 [3]. The disruption of TGFβ/Smad-dependent signaling during the progression of esophageal adenocarcinoma was confirmed by a report that showed Smad4 mRNA expression was progressively reduced in the metaplasia-dysplasia-adenocarcinoma sequence by promoter methylation [4]. In the same study, the authors demonstrated the loss of TGFβ-dependent induction of p21 and downregulation of mini-chromosome maintenance protein 2 in the majority of Barrett's tumorigenesis [4]. Interestingly, in a series of resected adenocarcinomas of the distal esophagus, *TGFB1* mRNA was expressed at significantly higher levels in tumor tissues compared to squamous epithelium and Barrett's mucosa. Additionally, univariant survival analysis has shown that *TGFB1* overexpression was associated with poor prognosis [5].

It is generally assumed that in esophageal metaplasia, the normal squamous esophageal epithelium undergoes transdifferentiation to resemble the columnar epithelium of the gastric tract and the intestine. BMP4, a member of the TGFβ family, has been shown to regulate the processes involved in this metaplastic transformation [6, 7]. The effects of BMP4 are tightly regulated by its natural antagonist, Noggin, which prevents the BMP-regulated development of the columnar epithelium in the esophagus during embryogenesis [8, 9]. BMPs, as well as another morphogen, sonic hedgehog, are typically not expressed in the normal adult esophagus [10], BMP4, however, has been shown to be re-expressed in esophagitis and Barrett's esophagus [6, 11]. Interestingly, sonic hedgehog can induce BMP4 secretion in stromal cells with myofibroblast morphology in response to acid injury [12].

Hedgehog signaling and epithelial-mesenchymal transition (EMT) have been implied in the morphogenesis of embryonic and adult tissues. When Hedgehog signaling is blocked, esophageal keratinocyte differentiation and squamous esophageal cancer cell invasion and growth are inhibited [13]. These findings suggest that the “mesenchymal gene expression” of undifferentiated cells is maintained or strengthened in cancer cells by Hedgehog-mediated signaling [13]. The analysis of other markers of EMT in gastroesophageal junction tumors has shown that the E-cadherin repressors Slug [14], Snail, and Twist [15] are associated with the malignant progression of esophageal adenocarcinomas. TGFβ is known to induce EMT through downregulation of E-cadherin and upregulation of mesenchymal markers [16].

A less studied member of the TGFβ family, the ligand Activin A, has been shown to be upregulated in the progression from Barrett's esophagus to dysplasia and ultimately esophageal adenocarcinoma [17]. When Activin signaling was inhibited with siRNA targeting the Activin A gene, *INHBA*, or with the Activin antagonist, Follistatin, esophageal adenocarcinoma cell lines demonstrated suppressed proliferation [17]. In a previous study analyzing Activin A function in esophageal squamous cell carcinoma, we showed that the effects of Activin A are largely context- and dose-dependent [18]. Herein, we describe the role of Activin A in Barrett's esophagus and esophageal adenocarcinoma cells, especially with respect to cell invasion and the crosstalk within the microenvironment.

## RESULTS

### The Inhibin β_A_ subunit of Activin A is increased in the progression to EAC

**Figure 1:**
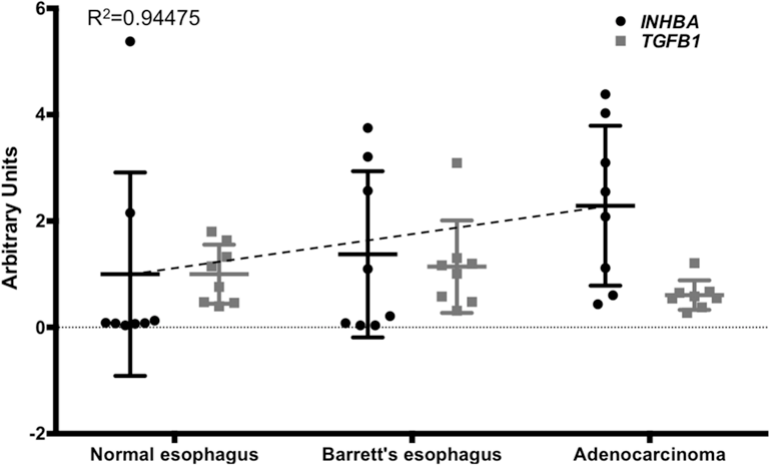
*INHBA* expression levels increase during the progression from normal esophagus to Barrett's esophagus and esophageal adenocarcinoma Comparison of *INHBA* and *TGFB1* expression was based on a publicly available GEO dataset (accession number GDS1321). Values for *INHBA* and *TGFB1* were measured from extracted and purified RNA, shown here as arbitrary units. A trend line for *INHBA* expression (dashed line) was calculated (*Y* = 0.6436x + 0.2666). *P*-values for *INHBA* normal vs. BE, *p* = 0.248; NE vs. EAC, *p* = 0.932; BE vs. EAC, *p* = 0.437.

TGFβ and Activin A have both been implicated in the pathology of esophageal adenocarcinoma [2–4, 17]. While TGFβ function is context-dependent, little is known about the role of Activin A in Barrett's tumorigenesis. We first queried publicly available dataset to investigate the expression of Activin A (a homodimer of Inhibin β_A_ subunits), TGFβ, and components of their signaling cascade during the progression from normal esophagus to esophageal adenocarcinoma. Analysis of 24 samples of normal squamous esophagus, Barrett's esophagus, and adenocarcinoma (*n* = 8 per group) for Activin A expression, encoded by the *INHBA* gene, showed a trending increase of expression during the progression to EAC (GDS1321, Figure 1). Interestingly, although previously shown to be involved in the subsequent metaplastic events, *TGFB1* expression remained unchanged (Figure 1). Expression of Inhibin A (*INHA*), an Activin A inhibitor formed through Inhibin β_A_ and Inhibin α subunit heterodimers and the antagonist Follitstatin, were also unchanged (Supplemental Figure S1). Analysis of other downstream targets of the signaling pathway showed that Smad2 and Smad3, as well as the common effector Smad4, were downregulated in the dataset used (Supplemental Figure S1). While these observations do not exclude TGFβ1 ligand function as an important factor in the biology of esophageal adenocarcinoma, they emphasize the significance of the components of these overlapping pathways, and led us to more closely investigate the role of Activin A signaling.

### Overexpression of Activin A (*INHBA*) in esophageal model cell lines results in cell type specific alterations of canonical and non-canonical pathways

**Figure 2:**
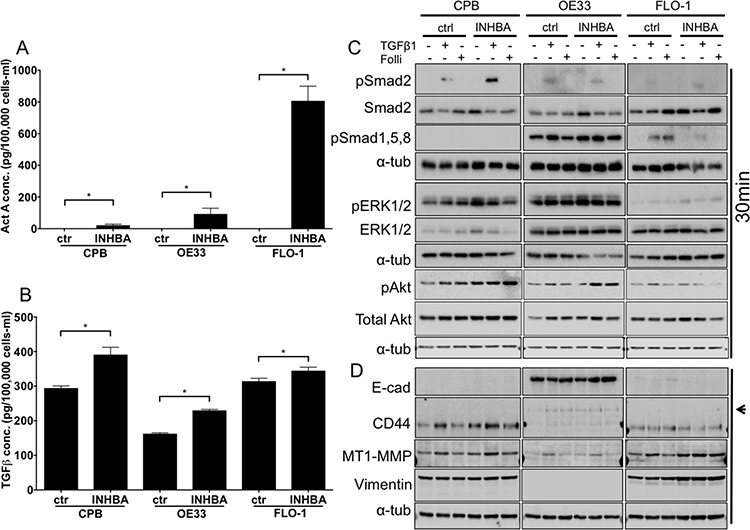
Overexpression of *INHBA* in esophageal model cell lines results in cell type specific alterations of canonical and non-canonical pathways **A.** Activin A concentration in conditioned media after overexpression of Activin A (*INHBA*) compared to empty vector control was assessed by ELISA, and normalized to the cell number at time of collection. Highest overexpression levels were achieved in FLO-1 cells. **B.** TGFβ concentration in conditioned media after overexpression of Activin A (*INHBA*) compared to empty vector control as assessed by ELISA. **C.** Protein expression of pSmad2, total Smad2, pSmad1,5,8, pERK1/2 and total ERK1/2, as well as pAkt and Akt was analyzed by Western blot. **D.** Antibodies against markers of epithelial-mesenchymal transition showed the expression of mesenchymal markers, such as standard form of CD44, high MT1-MMP and vimentin in CPB and FLO-1. OE33 cells expressed the variant CD44 isoform (arrowhead) and had high E-cadherin (E-cad). * *P* value < 0.05

Gene expression data from human tissue samples rarely allow insight into the cellular source of the RNA. As tumor samples are often comprised of epithelial tumor cells and stroma, the analyzed RNA is derived from both sources. To model epithelial Activin A overexpression, we chose the dysplastic cell line CPB and the EAC cell lines OE33 and FLO-1 and transduced them with an *INHBA* retroviral plasmid (two subunits of Inhibin β_A_ encoded by the *INHBA gene* result in the Activin A protein). *INHBA* overexpression was validated by ELISA in CPB, OE33 and FLO-1 cells. All three *INHBA*-overexpressing cell lines secreted significantly higher levels of Activin A compared to control (Figure 2A). Interestingly, when normalized to the number of cells at the end of the collection period (48 hours), Activin A concentration was higher in OE33 *INHBA* cells than CPB *INHBA* cells, while FLO-1 *INHBA* cells secreted the highest levels of Activin A overall. To identify if *INHBA* overexpression affected TGFβ1 secretion levels, we performed ELISA to measure TGFβ1 in the conditioned media. Levels of secreted TGFβ1 significantly increased in the *INHBA*-overexpressing cells compared to control (Figure 2B). As the function and availability of Activin A can be regulated by secreted factors, such as the antagonists Follistatin and Inhibin A, we also measured the concentrations of Follistatin (pan-antibody recognizing all three Follistatin isoforms FS288, FS300 and FS315) and Inhibin A in the collected conditioned media by ELISA. The levels of these factors were below the limit of assay sensitivity when compared to the positive controls (data not shown).

To identify which downstream signaling targets were activated in response to *INHBA* overexpression, we collected protein lysates of untreated cells, as well as cells treated with recombinant TGFβ1 as a positive control and Follistatin-288. Smad2, a downstream target of Activin A and TGFβ phosphorylated upon signal transduction, was not activated in any of the *INHBA*-overexpressing cell lines, leading us to conclude that continuous exposure to Activin A desensitizes the cells to signal induction (Figure 2C). Stimulation with TGFβ1 as a control, however, elicited phosphorylation of Smad2 within 30 minutes. TGFβ1 stimulation resulted in phosphorylation of Smad2 in OE33 and FLO-1 cells. We also analyzed phosphorylation of Smad1,5,8, which is typically induced by BMP2 and BMP4. Interestingly, OE33 cells, which express more epithelial markers and less mesenchymal markers than CPB and FLO-1 cells (Figure 2D), had high baseline phosphorylation of Smad1,5,8 (pSmad1,5,8) and pERK1/2. pSmad1,5,8 was increased by TGFβ1 in both control and *INHBA* expressing OE33 cells, and overall *INHBA*-overexpressing OE33 cells showed the strongest signal for pSmad1,5,8 among the three cell lines. In FLO-1 cells pSmad1,5,8 was suppressed by *INHBA* overexpression, but present in control cells treated with TGFβ1 or Follistatin-288. FLO-1 cells showed no activation of the ERK pathway. CPB cells, which showed no pSmad1,5,8 phosphorylation, had a strong signal for pERK1/2. pAkt levels were similar amongst control and *INHBA* overexpressing cells and were not altered with any of the treatments.

Analysis of EMT markers showed a lack of E-cadherin in CPB and FLO-1, which generally exhibit a more mesenchymal phenotype than OE33 cells. OE33 typically show a cobblestone growth appearance and express high levels of E-cadherin. Accordingly, CPB and FLO-1 cells expressed the mesenchymal markers vimentin and MT1-MMP, as well as the mesenchymal variant of CD44. Upon *INHBA* overexpression, we observed increased levels of CD44 and MT1-MMP in CPB cells, which were further enhanced 48 hours after TGFβ1 stimulation. While the mesenchymal variant of CD44 is not expressed in OE33 cells, the standard form is (arrowhead to upper band, [19, 20]). MT1-MMP, a membrane anchored matrix metalloprotease, was also increased in response to TGFβ1 in FLO-1 cells and upregulated in *INHBA*-expressing FLO-1 cells. CD44 is expressed at similar levels in the FLO-1 empty vector and *INHBA* cell lines. *INHBA* overexpression resulted in higher levels of vimentin in FLO-1 cells compared to empty vector control (Figure 2D). E-cadherin expression levels were largely unchanged in OE33 cells.

To identify if changes in the receptor complex components occur in these cells lines, we analyzed the expression of TGFBR2, ACVR1B, ACVR2, ACVR2B and the common downstream target Smad4. Aside from low levels of Smad4 in OE33 and low TGFBR2 in FLO-1 cells, all components were present in the analyzed cells (Supplemental Figure S2A).

In conclusion, we assessed the contributions of Smad-dependent canonical and non-canonical signaling targets, and showed phosphorylation of Smad2 only in response to TGFβ1, leading us to assume that *INHBA* overexpression does not alter baseline activation of Smad2. CPB *INHBA* cells had a stronger pSmad2 signal than control cells when treated with TGFβ1. We thus conclude that overexpression of *INHBA* in pre-malignant cells may increase the sensitivity to TGFβ1 signaling. Non-canonical pathway induction was measured by phosphorylation of ERK1/2 and Smad1,5,8, showing pSmad1,5,8 to be activated in OE33 and increased in the presence of *INHBA* overexpression, yet downregulated in FLO-1 *INHBA* cells upon TGFβ1 stimulation as well as with Follistatin inhibition when compared with respective FLO-1 vector controls.

### *INHBA* overexpression increases cell invasion in CPB and FLO-1, which exhibit mesenchymal features

Next, we assessed functional alterations of the *INHBA*-expressing cell lines compared to their control cells. To analyze growth rates in response to *INHBA* overexpression, we performed WST-1 viability assays over 96 hours and showed that overexpression of *INHBA* increased proliferation compared to control empty vector cells in all three cell lines (Supplemental Figure S2B–S2D). Addition of Follistatin-288 during the incubation period decreased growth by 96 hours. Interestingly, an Activin A neutralizing antibody could not inhibit growth, indicating that the functional effects of Follistatin could be mediated by other targets. Folllistatin has been shown to bind with low affinity to several other members of the TGFβ superfamily, including growth and differentiation factor-9 (GDF9), myostatin (GDF8), and several of the bone morphogenetic proteins (BMPs) [21, 22]. Although Follistatin does not bind to TGFβ1 or 2, binding to TGFβ3 has been reported [23].

**Figure 3:**
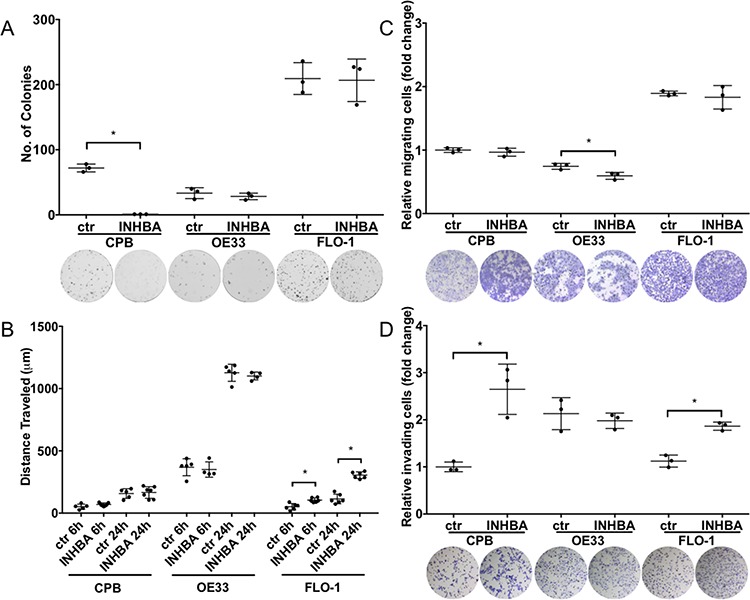
*INHBA* overexpression impacts colony formation, migration and invasion potential in a cell-type specific manner **A.** *INHBA* overexpression inhibited colony formation of CPB cells compared to control (ctr), but not in the esophageal adenocarcinoma cell lines, OE33 and FLO-1. **B.** Scratch assays showed high migration capabilities for OE33 cells, independent of *INHBA* status. *INHBA* overexpression enhanced scratch closure in FLO-1 cells (FLO-1 INHBA). **C.** Migration measured in transwell chamber assays towards a full medium gradient showed inhibition of OE33 migration after *INHBA* overexpression (OE33 *INHBA*). **D.** CPB *INHBA* and FLO-1 *INHBA* had an increased invasion potential compared to empty vector control cells (ctr) when grown in Boyden chamber invasion assays. OE33 *INHBA* invasion was equal to empty vector control (ctr). Statistical analysis was performed using Student's *t*-test, *p < 0.05.

Using colony formation assays to examine the cell survival and self-renewal capacities of *INHBA*-expressing cells, we detected reduced colony formation in CPB cells; however, no effect on OE33 and FLO-1 cells was observed (Figure 3A). The response of CPB cells to *INHBA* overexpression indicated that premalignant cells or cells at an early stage of EAC progression reduce their capability for cell survival or self-renewal while cancer cell lines evade a Activin A-mediated response.

To investigate the migratory and invasive potential of these cells, we employed two approaches: one that would account for gradient-independent migration, the scratch assay, and the other the chemotaxis-dependent Boyden chamber assays (Figures 3B–3D). Of the tested cell lines, OE33 cells had the greatest migratory potential in scratch assays, yet no differences between *INHBA*-expressing and control cells were observed (Figure 3B). However, cell migration in scratch assays was increased in *INHBA*-expressing FLO-1 cells (Figure 3B). Migration in Boyden chamber assays with full media as a chemoattractant showed no changes in migration for CPB and FLO-1 *INHBA* and control cells. *INHBA* overexpression reduced Boyden chamber migration of OE33 cells (Figure 3C). Invasion assays, in which cells have to digest a Matrigel matrix in a MMP-dependent process, demonstrated significantly higher invasion in *INHBA*-expressing CPB and FLO-1 cells, but not OE33 cells (Figure 3D). As we have shown higher levels of CD44 and MT1-MMP in CPB and FLO-1 cells, the increased cell invasion could be dependent on the expression of these mesenchymal markers and the greater capability for digesting the matrix. Follistatin-288 and Activin A neutralizing antibody were used as additional controls in this assay to demonstrate the specificity of the function of Activin A in cell invasion (Supplemental Figure S3). The antagonist Follistatin reduced cell invasion in control and *INHBA*-overexpressing cells, but the Activin A neutralizing antibody had an even greater effect (Supplemental Figure S3). This observation is interesting in light of the greater efficacy of Follistatin in reducing cell growth compared to the neutralizing antibody (Figure 2B–2D). We postulate that the corroboration of different pathways controls the diverse functions of proliferation and invasion.

### Stimulation with exogenous Activin A results in downstream activation of canonical and non-canonical pathway components

**Figure 4:**
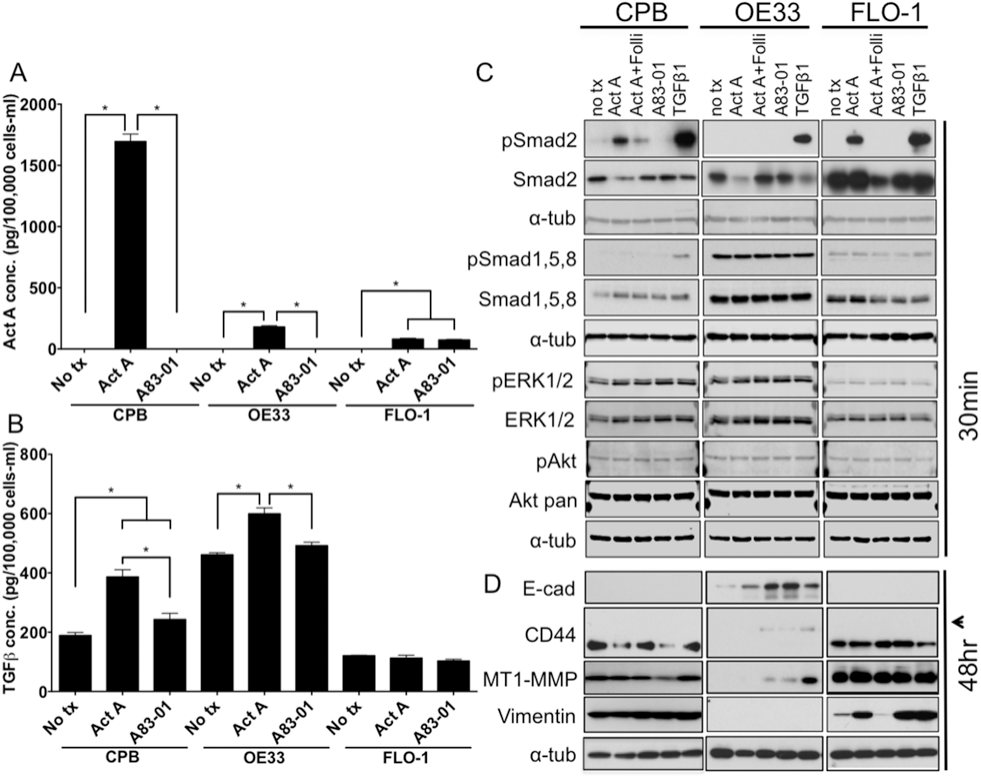
Stimulation with recombinant Activin A regulates TGFβ1 secretion and induces canonical downstream signaling in CPB and FLO-1 cells **A.** Activin A concentration in conditioned media after stimulation with recombinant Activin A (Act A) or the TGFβ receptor inhibitor A83-01, as assessed by ELISA and normalized per 100,000 cells after 48 hour incubation. **B.** TGFβ1 concentration in conditioned media after stimulation with recombinant Activin A or the TGFβ receptor inhibitor A83-01, was assessed by ELISA. TGFβ1 concentration was increased in response to stimulation with recombinant Activin A and reduced with A83-01 in CPB and OE33, but not in FLO-1. Concentrations were normalized to cell number at time of collection. **C.** Antibody against phospho-Smads (pSmad2; pSmad1,5,8) and total Smad2, as well as pERK1/2 and pAkt compared to total ERK1/2 and Akt were used for Western Blot analysis. pSmad2 is induced in response to Activin A and TGFβ1 in CPB and FLO-1, but only with TGFβ1 in OE33. **D.** Antibodies against markers of epithelial-mesenchymal transition showed increased expression of the variant form of CD44 (arrowhead) in epithelial OE33 cells following stimulation with Follistatin (Folli) and Activin A, as well as TGFβ1. MT1-MMP was increased mainly with recombinant TGFβ1 in OE33. FLO-1 cells, which exhibit a mesenchymal phenotype, had a further increase in vimentin expression with Activin A, but also with addition of A83-01 and TGFβ1. Statistical analysis was performed using ANOVA, *p < 0.05.

To explore the differences between paracrine and autocrine signaling effects, we next added recombinant Activin A to the culture media. In this set of experiments, we aimed to investigate if the model cell lines exhibit differential responses when stimulated with recombinant Activin A, a model of non-epithelial-derived Activin A. Using ELISA, we measured Activin A and TGFβ concentrations in conditioned culture media collected from untreated cells, cells treated with recombinant Activin A, or cells treated with the TGFβ receptor inhibitor A83-01 (Figure 4A, 4B). Activin A levels were found to be higher in Activin A-stimulated CPB cells than in OE33 and FLO-1 cells (Figure 4A). As the concentration of Activin A, when normalized to the amount of cells at the end of the 48-hour incubation period concentration, was much lower than expected in the OE33 and FLO-1 cells, we speculate that the availability is lower either due to decreased stability or increased uptake by the cells. The TGFβ1 concentration in the conditioned media was increased upon exogenous Activin A stimulation in CPB and OE33 cells, but not FLO-1 cells (Figure 4B). The addition of A83-01 had no effect on TGFβ1 secretion in in all three cell lines.

Western Blot analysis showed no phosphorylation of Smad2 in any of the three cell lines tested when untreated (Figure 4C). Stimulation with Activin A induced phosphorylation of Smad2 in CPB and FLO-1 cells, but not in OE33 cells. TGFβ1, which was used as a positive control for the induction of Smad2 phosphorylation, resulted in robust Smad2 activation across all tested cell lines. pSmad1,5,8 was detected at high levels in OE33 cells independent of Activin A stimulation or inhibition. CPB and FLO-1 cells showed increased pSmad1,5,8 only upon TGFβ1 stimulation. pERK1/2 and pAKT remained unchanged in response to the different treatments. Again, overall pERK1/2 was higher in CPB and OE33 cells compared to FLO-1, similar to our observations in the *INHBA* overexpression model (Figure 2C). The epithelial marker E-cadherin was expressed in OE33 and increased expression was detected upon inhibition of Activin A with Follistatin or TGFβ1 inhibition by A83-01. CPB and FLO-1 expressed the standard form of CD44, while OE33 expressed the variant form, which is commonly associated with a more epithelial phenotype (arrowhead, Figure 4D). MT1-MMP has the highest expression in FLO-1 cells. While CPB cells also expressed MT1-MMP, OE33 only showed a signal after TGFβ1 stimulation. Both CPB and FLO-1 had expression of the mesenchymal marker vimentin, which was absent in OE33 cells. Interestingly, Activin A increased vimentin expression in FLO-1 cells, which was reduced after the addition of Follistatin, demonstrating specificity of the Activin A-mediated effect (Figure 4D). Analysis of the receptor expression confirmed the low levels of TGFBR2 in FLO-1 cells (Supplemental Figure S4A). Overall, TGFBR2 expression levels were unaffected by stimulation with Activin A or by the use of antagonists and inhibitors, but upon TGFβ1 stimulation TGFBR2 expression was downregulated in CPB cells, possibly due to degradation upon signaling induction. The unchanged expression levels for the signaling receptors are not unexpected as the regulation of receptor function depends mostly on phosphorylation events, localization within the cells, and endocytosis upon ligand binding to ensure recycling of the receptors. Yet deletion, mutation, and epigenetic silencing can lead to the loss of expression and, therefore, evasion of a cytostatic response has been reported in the literature [24].

### Activin A stimulation increased cell invasion in OE33 cells

As the stroma can be a major source of chemokines and cytokines regulating tumor growth and invasion, we measured the effects of recombinant Activin A treatment on cell viability (Supplemental Figure S4B–S4D), colony formation, cell migration (scratch assay), and chemotaxis-dependent cell migration and invasion (Boyden chamber). When we examined cell growth after treatment with recombinant Activin A, Follistatin, an Activin A neutralizing antibody, and combinations of Activin A with the respective antagonists, we observed largely unchanged cell growth with the different conditions (Figure S4B–S4D). CPB cells showed an increase after treatment with Follistatin, but not neutralizing antibody (Figure S4B). OE33 cells exhibited a decrease in growth in the presence of Activin A together with Follistatin. FLO-1 cells, while unresponsive to Activin A stimulation, showed increased cell growth in the presence of Activin A and its antagonists (Figure S4D). These results are in contrast to the overall induction of proliferation in all cell lines after *INHBA* overexpression (Figure 2B).

**Figure 5:**
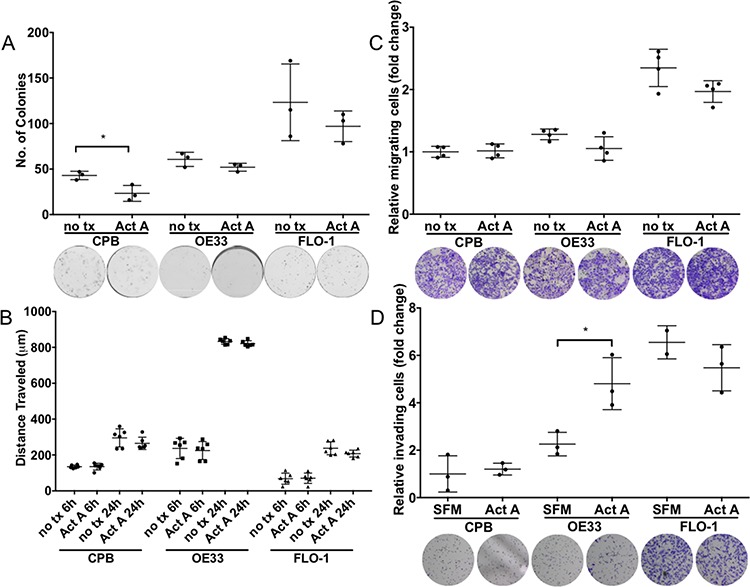
Stimulation with recombinant Activin A affects colony formation, migration and invasion potential in a cell-type specific manner **A.** Activin A overexpression inhibited colony formation of CPB cells, but not in esophageal adenocarcinoma cell lines, OE33 and FLO-1. **B.** Scratch assays showed high migratory capabilities for OE33 cells. **C.** Migration measured in transwell chamber assays towards a full medium gradient showed no effect from Activin A stimulation. **D.** Using Activin A as a chemoattractant increased the invasive capability of OE33 cells when measured in Boyden chamber invasion assays (SFM: serum-free medium; Act A: serum-free medium with Act A). *t*-test is used to calculate significance, *P* value < 0.05.

Similar to the data observed with *INHBA* overexpression, colony formation was reduced in CPB cells in the presence of recombinant Activin A (Figure 5A). Activin A, however, had no effect on the EAC lines OE33 and FLO-1 (Figure 5A), indicating that Activin A exerts colony reduction in dysplastic but not tumor cells.

When we studied the response to Activin A in scratch assays, we found that while OE33 had the highest migratory potential in the scratch assays, Activin A stimulation had no effect compared to untreated controls (Figure 5B). No significant differences were observed between untreated and Activin A treated cells in chemotaxis migration (Figure 5C), however, cell invasion assays using Activin A as a chemoattractant increased OE33 cell invasion (Figure 5D). When Follistatin in combination with Activin A was used, FLO-1 cells demonstrated a diminished potential for invasion, indicating a dependence on signaling targeted by Follistatin inhibition, such as Activin A, and potentially other pathways, such as BMP. Other treatments including neutralizing antibody surprisingly had no effect on cell invasion (Supplemental Figure S5), possibly hinting at the necessity for additional growth factors to contribute to this phenotype. Fetal bovine serum as a positive control elicited considerable invasion in all three cell lines.

In all, Activin A concentrations in the ELISA assay (Figure 4A) identified that FLO-1 cells had the lowest measurable Activin A concentration 48 hours after treatment and therefore the lack of response in the functional assays could be dependent on Activin A availability. Interestingly, Activin A media concentrations were lower for OE33 cells than CPB after Activin A stimulation, yet cell invasion could be induced in OE33 cells.

Overall, comparison of the results for Activin A stimulation and the retroviral overexpression of *INHBA* suggest that the Barrett's cell line CPB is responsive to Activin A as a modulator of cell survival, which ultimately results in reduced colony formation. However, only overexpression of *INHBA* enhances cell invasion in CPB and FLO-1 cells, while stimulation with recombinant Activin A does so for OE33 cells. A limitation of the approach comparing overexpression as a model for autocrine and stimulation for paracrine signaling is not only the variability in ligand availability and dosage, as can be seen by the differences in concentration by ELISA, but also the long-term vs. acute exposure to the ligand. It appears however, that while Activin A stimulation (acute response) can elicit functional changes, only overexpression can result in intrinsic cellular changes, which ultimately becomes the driver for events such as cell invasion after long-term exposure (Figure 3). While the stroma as a source of Activin A seems to effect migration and invasion less than autocrine functions of tumor-secreted Activin A, the elicited functional responses are potentially influenced by acute vs. chronic exposure and crosstalk with other pathways. This hypothesis is supported by the variable regulation of Activin A-dependent functions in the presence of Follistatin or Activin A neutralizing antibody, but will require additional experimentation.

### Columnar keratins are differentially expressed in organotypic reconstruct cultures

**Figure 6:**
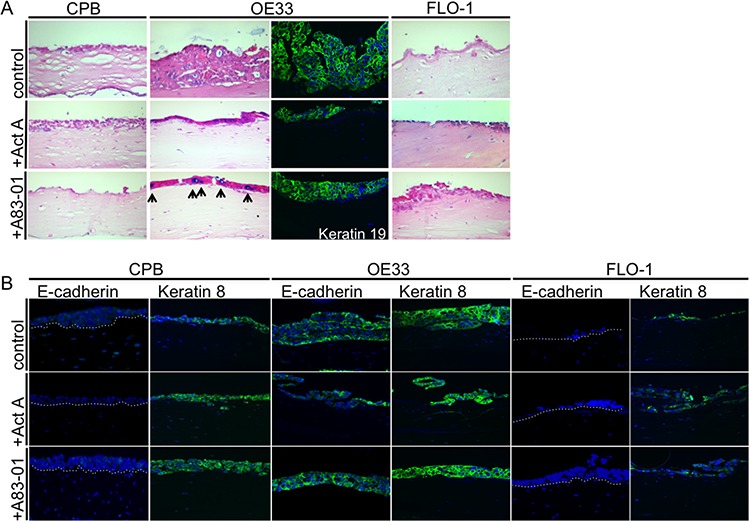
Barrett's esophagus and esophageal adenocarcinoma cells in organotypic reconstruct cultures **A.** OE33 cells had increased Alcian Blue staining upon A83-01 treatment (arrows). OE33 cells were also positive for the columnar marker cytokeratin 19 (keratin 19). **B.** CPB and FLO-1 cells, which have a mesenchymal phenotype, express no E-cadherin, but OE33 showed positive E-cadherin staining by immunofluorescence. Anti-keratin 8 antibody showed that the signal increased in CPB following stimulation with Activin A or treatment with A83-01. Keratin 8 signal was weaker in FLO-1 cells in the presence of A83-01.

The previous assays allow the analysis of epithelial cells *in vitro*, but these methods do not take into account for epithelial cell crosstalk with matrix components and mesenchymal cell types. For that reason, we grew CPB, OE33, and FLO-1 cells in the presence and absence of Activin A in organotypic cultures on a collagen/Matrigel matrix with embedded fibroblasts to mimic a physiological stromal context. CPB and FLO-1 cells, which have a mesenchymal phenotype, were unable to form a multilayered epithelium due to the lack of cell-cell adhesion. However, stimulation of the culture with Activin A, as well as inhibition of TGFβ with A83-01, induced overall epithelium formation in CPB and FLO-1 cells. Both cell types were negative for Alcian Blue staining, which was used to detect mucin-secreting cells, and the columnar marker cytokeratin 19 (CK19) (Figure 6A). The epithelial adenocarcinoma cell line OE33 exhibited Alcian Blue-positive cells, indicating the presence of mucin secreting goblet cells (arrows, Figure 6A), and positive staining for CK19 (Figure 6A) in Activin A-treated and control organotypic cultures.

When stained for E-cadherin and for the columnar keratin, cytokeratin 8 (CK8), CPB cells showed no signal for E-cadherin, validating the Western Blot data (Figure 4E). CK8, however, could be detected and increased following treatment with Activin A and A83-01 (Figure 6B). OE33 cells were positive for both E-cadherin and CK8, independent of the treatment conditions. No E-cadherin staining could be detected in FLO-1 cells, and only a few cells of the epithelium were positive for CK8 (Figure 6B). Cytokeratin 14, a squamous marker, was not detected by immunofluorescence (data not shown).

Columnar cytokeratins CK8 and CK19 are expressed in glandular, non-squamous epithelium, including Barrett's Esophagus [25], and therefore demonstrate the shift towards a columnar phenotype compared to normal squamous esophageal tissue.

### Activin A regulates SOX9 nuclear localization

**Figure 7:**
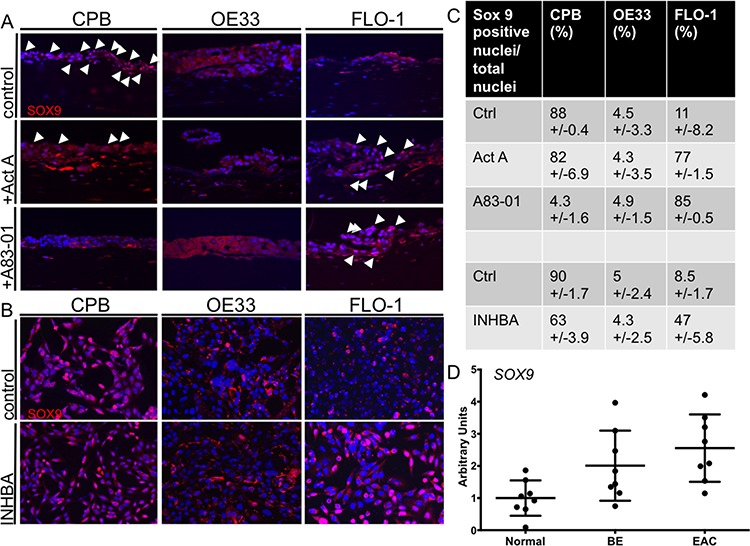
Activin A induces nuclear SOX9 in FLO-1 cells **A.** In organotypic cultures, CPB cells showed nuclear SOX9 (arrowheads) with and without Activin A (Act A) stimulation; the signal was reduced with A83-01. OE33 cells show no nuclear SOX9 in any of the conditions. The intensity for SOX9 staining was low in FLO-1 control cells with no nuclear localization, but increased with Activin A (Act A) and A83-01, as shown by the white arrowheads. **B.** In monolayer, CPB control and INHBA overexpressing cells had a nuclear SOX9 signal. OE33 cells showed no nuclear SOX9 in any of the conditions. The intensity for SOX9 staining was low in control cells with no nuclear localization in FLO-1 cells, but increased in FLO-1 INHBA. **C.** SOX9-positive nuclei were counted and calculated as percentage per total nuclei per field. Four fields per replicate image were counted. **D.** The publicly available GEO dataset (accession number GDS1321) was used for analysis of SOX9 expression and showed overall increased SOX9 expression in the progression from Barrett's esophagus to esophageal adenocarcinoma.

We further aimed to determine the expression of SOX9 in response to Activin A signaling. SOX9 is known to drive columnar differentiation of the esophageal squamous epithelium [26] and has been described to attribute stem cell-like properties to esophageal cancer cells [27]. We hypothesized that because mesenchymal cells have more stem cell-like features, SOX9 should be detected with higher frequency in the nuclei of CPB and FLO-1 cells. To address the expression and localization of SOX9 in response to Activin A and TGFβ signaling, we performed immunofluorescence staining with an anti-SOX9 antibody on the organotypic cultures. In untreated cultures, CPB cells were positive for nuclear SOX9, while few OE33 and FLO-1 cells were positive (white arrowheads, Figure 7A). Upon stimulation with Activin A, SOX9 expression was unaffected in CPB cells, though the number of SOX9-positive nuclei in the fibroblasts increased. Organotypic cultures of OE33 cells showed increased positive SOX9 signal in the fibroblasts, but not in the OE33 cells themselves. Increased nuclear staining was observed in FLO-1 cells and the surrounding fibroblasts after Activin A stimulation (white arrowheads, Figure 7A). TGFβ inhibition by A83-01 decreased nuclear SOX9 in the CPB cultures, but did not inhibit SOX9 nuclear localization in the FLO-1 cells (Figure 7C). Interestingly, when *INHBA*-overexpressing cells were grown on plastic, nuclear SOX9 was also high in CPB and low in OE33 cells, but SOX9-positive nuclei were less frequent in CPB *INHBA* cells than in the Activin A-stimulated cells grown in organotypic cultures (Figure 7B). In FLO-1 cells, which showed an increased number of SOX9-positive nuclei upon Activin A and A83-01 treatments, we still detected an increase in SOX9-postitive nuclei in *INHBA*-overexpressing cells (Figure 7B), but not to the same extent as in cultures treated with recombinant Activin A (Figure 7C). These observations show that expression and nuclear SOX9 localization in CPB cells are TGFβ signaling–dependent, demonstrated by suppression of TGFβ signaling by A83-01 in organotypic culture. Conversely, the *INHBA*-dependent increase of nuclear SOX9 in FLO-1 cells is not reversible through inhibition of TGFβ by A83-01. Differences in numbers between organotypic cultures and plastic indicate a role of the stroma in the regulation of the signaling pathways resulting in SOX9 activation. Using the same dataset as in Figure 1, we showed an increase in SOX9 expression during the progression from Barrett's esophagus to esophageal adenocarcinoma (Figure 7D). Together, we believe that these data indicate the importance of the microenvironment for signal transduction and SOX9 protein expression.

## DISCUSSION

Esophageal adenocarcinoma (EAC) has a poor patient outcome, with a 5-year survival of only 14% [28]. A recognized risk factor for EAC is Barrett's esophagus, which has been shown to progress from a pre-malignant lesion to EAC in 5–10% of cases [29]. EACs can arise rapidly in patients diagnosed with Barrett's esophagus, even under careful surveillance. It is thought that the transformation of non-dysplastic to dysplastic Barrett's esophagus, and ultimately esophageal adenocarcinoma, is driven by a stepwise accumulation of mutations or specific oncogenic events that underlie progression. Yet, most of the commonly mutated genes in EAC—except *TP53* and *SMAD4—*have also been found in non-dysplastic Barrett's esophagus that did not progress towards cancer [30]. These findings and the fact that targeted therapies brought little improvement due to reactivation of the targeted pathway, hyperactivation of alternative pathways, and cross-talk with the microenvironment [31] highlight that aside from genomic catastrophes, such as gene inactivation through chromosomal rearrangements and telomere integrity [32, 33], other important mechanisms are at play in EAC progression.

We have focused here on the activation of canonical and non-canonical signaling by Activin A to identify the contributions of this TGFβ family member to the pathology of EAC.

### Signaling

Activins and BMPs are classified as members of the TGFβ superfamily, and bind to related transmembrane receptors, resulting in overlap in their intracellular signaling cascades [34] and downstream function [35–38]. This has been shown by deletions of TGFβ2, TGFβ3 and Inhibin β_A_, which all result in cleft palate defects in the respective mouse model. Similar to a 10-bp polyadenine tract within the TGFβ receptor type II gene (*TGFBR2)* that is prone to frameshift mutations in gastrointestinal cancers, mutations in *ACVR2* have been identified in colorectal and pancreatic cancers [39]. Additionally, *ACVR1B* is commonly mutated in pancreatic cancer [40], and in a majority of sporadic colorectal cancers BMPR2 expression is impaired [41]. Conversely, BMP and Activin Membrane-bound Inhibitor, BAMBI, is upregulated in colorectal cancer and is under direct regulation of the Wnt pathway, [42] a component of a gene expression profile that predicts metastasis [43]. Exome and whole-genome sequencing of EAC has identified recurrent driver events with high frequency, such as mutations in *TP53* and *CDKN2A*, but also *PTEN* and *SMAD4* [44, 45]. Interestingly, Activin A, which canonically signals through the Smad cascade, but utilizes a different set of receptors (ACVR2A or ACVR2B), was able to activate Smad2 in CPB and FLO-1 cells, but failed to do so in OE33 cells. OE33 cells, on the other hand, showed overall non-canonical activity of the MAPK/ERK pathway, as well as activation of BMP signaling as measured by pSmad1,5,8, the latter being increased upon *INHBA* overexpression. This could indicate that these signaling pathways are somewhat promiscuous and that ligands can utilize undesignated receptors [46, 47]. Furthermore, stimulation with TGFβ1 upon overexpression of *INHBA* resulted in activation of the canonical and non-canonical pathways (for OE33 and FLO-1 cancer cells). TGFβ1 has been shown to induce phosphorylation of Smad1,5,8 in endothelial cells mediated by TGFBR1 and ACVL1 [47]. Interestingly, although functional consequences, such as a decrease in clonogenicity, were observed in both acute and long-term exposure settings for Barrett's esophagus cells, we could not measure meaningful canonical and non-canonical signaling activation in *INHBA* overexpressing cells. This could be potentially due to an acquired insensitivity after prolonged exposure, yet we observed increased cell invasion, possibly due to intrinsic cell changes.

### Tumorigenicity and invasion

In esophageal squamous cell carcinoma, Activin A has been associated with tumor aggressiveness and increased MMP-7 expression. This increase in aggressiveness is accompanied by increased proliferation [48] and MMP-7 activity [49]. However, when the microenvironment is taken into account, the regulation of Activin A through its inhibitor Follistatin or TGFβ receptor inhibitor has differential effects when keratinocytes and fibroblasts were grown in organotypic cultures [18]. Our previous study showed that while cell invasion was increased upon Activin A stimulation in a premalignant cell model, inhibition of Activin A and TGFβ1 further enhanced cell invasion. Based on the importance of the balance between Activin A/TGFβ and BMP signaling, squamous epithelial homeostasis appears to be regulated by fine-tuning the concentrations and activity of the different growth factors and their functions [18]. Here we show that activation of Activin A signaling results in decreased cell migration of OE33 cells in the context of autocrine signaling (Boyden chamber), yet increased OE33 cell invasion upon stimulation with Activin A, suggesting a stromal contribution. Invasion of CPB and FLO-1 cells was increased upon *INHBA* overexpression, but was not affected by stimulation. Given the variability in Activin A concentration between overexpression and recombinant stimulations in the FLO-1 cells, the results could be dependent on a lower concentration of Activin A. Overall, we speculate that the source of Activin A elicits different cellular responses in a context-dependent manner. Aside from the source, an important consideration is the amount of Activin A available, as sudden influx and receptor occupancy in an acute setting will activate signaling; yet during long-term exposure, low-affinity ligand receptor interactions could lead to more promiscuity.

The microenvironment has been recognized as playing an increasingly important role in carcinogenesis. Gene ontology analysis has identified a strong inflammatory component in Barrett's tumorigenesis, and key pathways included are cytokine-receptor interactions and TGFβ [50, 51]. We extrapolate from our data that the endogenous production of Activin A may result in different phenotypic and functional outcomes than the mimicked paracrine stimulation of the cells. At the same time, similar to the differential effects on multiple cell lines as described above, we found profound variation when analyzing models of EAC progression between dysplastic cells (CPB) and esophageal adenocarcinoma cell lines with an epithelial (OE33) or mesenchymal phenotype (FLO-1) and their responses to Activin A.

Wang et al. have shown that squamous-to-columnar metaplasia can occur when bile-induced injury reactivates latent developmental pathways [52]. Hedgehog signaling in squamous epithelial cells upon bile-induced injury stimulated stromal expression of BMP4 by esophageal fibroblasts, resulting in epithelial SOX9 expression [52]. Given the activation of pSmad1,5,8, the fine balance between Activin A and BMP signaling might be regulating the context-dependent functional outcomes.

### Stemness and resistance

Activin A has been identified as necessary for the maintenance of self-renewal in human embryonic stem cells through the induction of Oct4, Nanog, Nodal, Wnt3, but more importantly the induction of basic FGF and the suppression of BMP [53]. These data indicate the role of Activin A as a mediator of stemness and potentially as a cancer stem cell marker. Suppression of the downstream target Id2 by Activin A and TGFβ is central in the induction of EMT [54], which is antagonized by BMP. Supporting the important role in regulating self-renewal of stem cells and cell-fate determination in the initiation and progression of Barrett's esophagus to EAC, it has been shown that in the normal esophagus, small clusters of Oct3/4-positive cells are nested in the basal cell layer, representing a pool of progenitor cells. Concomitant with the activation of Notch and TGFβ signaling in esophageal adenocarcinoma, an expansion of the Oct3/4 positive cell clusters can be observed [2]. SOX proteins, documented as stem cell markers, also exhibited increased expression in esophageal adenocarcinoma cells [2].

SOX9 has been implicated in the induction of a cancer stem cell phenotype in esophageal cancer [2, 26]. Expression of SOX9 in squamous epithelial cells has been shown to induce the formation of a columnar-like epithelium with the expression of columnar differentiation markers such as cytokeratin 8, demonstrating that columnar dedifferentiation and expression of intestinal markers reminiscent of Barrett's esophagus can be driven by SOX9 [26]. The involvement of bile reflux injury in the context of Barrett's tumorigenesis has been shown in acid treatment experiments using normal esophageal squamous, OE33 cells, and a mouse model of bile reflux. Acid or bile exposure led to an induction of stromal BMP4 and epithelial SOX9 resulting in conversion from squamous to columnar epithelium along with the expression of columnar cytokeratins [55]. Nuclear SOX9 is also detected in a surgical model of reflux by esophagojejunostomy [56]. Additional support that SOX9 may be an important early event in the development of Barrett's tumorigenesis is seen in the activation of SOX9 following loss of β2-spectrin, which induces a TGFβ signaling switch from tumor suppressor in normal cells to tumor promoter in fibroblasts and EACs [27]. Upstream of SOX9, YAP1 has been shown to be a major determinant of cancer stem cell properties in non-transformed and esophageal cancer cells. YAP-induced upregulation of SOX9 was concomitant with the acquisition of stem cell properties [57].

Our data show that induction of nuclear SOX9 is potentially associated with a mesenchymal phenotype, as epithelial OE33 cells are negative for SOX9. Activin A stimulation, as well as its overexpression, resulted in increased nuclear SOX9 localization and may be associated with stem cell-like features, such as the expression of EMT markers. Stem cell-like properties have been attributed to the mediation of therapy resistance [58]. Kim Ah et al. [59] reported that, in response to ionizing radiation, TGFβ downregulates *c-Myc* mRNA expression and inhibits the growth of OE33 EAC cells *in vitro*. While TGFβ enhanced radioresistance of OE33 cells, it did not affect the radiosensitivity of squamous carcinomas KYSE and OE21. The TGFβ-enhanced radioresistant phenotype was associated with induced G0/G1 cell cycle arrest and upregulation of the G1 cyclin-dependent kinase inhibitor p27kip1, as well as downregulation of c-Myc protein expression. Interestingly, conditioned medium obtained from unirradiated OE33 cells enhanced radioresistance compared with fresh medium. This enhancement was abrogated by pre-incubation of conditioned medium with a neutralizing anti-TGFβ antibody, suggesting endogenous TGFβ production by OE33 cells. Given the reports of submucosal metaplasia after Barrett's esophagus radioablation, it remains to be seen if SOX9 expression coupled with high Activin A serum levels would be useful as an early detection marker.

Taken together, we aimed to determine the role of tumor-derived and stromal Activin A during sequential events of esophageal transdifferentiation promoting Barrett's tumorigenesis. We demonstrated that aside from known mutational or epigenetic alterations, activation of signaling is pleiotropic and context-dependent, thereby highlighting the complex crosstalk with the microenvironment.

## MATERIALS AND METHODS

### Cell culture

The Barrett's esophagus cell line CPB (CRL-4028) was purchased from American Type Culture Collection (ATCC) and cultured with epithelial cell medium 2 (ScienCell, Carlsbad, CA) supplemented with 5% fetal bovine serum (FBS, Hyclone, GE Healthcare, Pittsburgh, PA) and antibiotics, 100 units/mL penicillin and 100 μg/mL streptomycin (Gibco, Carlsbad, CA). The esophageal adenocarcinoma cell lines, OE33 and FLO-1, were derived by Dr. David Beer [60] and grown in RPMI and DMEM (Invitrogen, Carlsbad, CA), respectively, with 10% FBS at 37°C in 5% CO_2_. Fibroblasts were grown in DMEM with 5% FBS (Hyclone), 100 units/mL penicillin, and 100 μg/mL streptomycin (Gibco). For treatment with growth factors 5 ng/ml recombinant human TGFβ1, 10 ng/ml Activin A, 100 ng/ml Follistatin-288, 100 ng/ml Nodal (all R&D Systems, Minneapolis, MN Systems) or 1 μM A83-01 (Tocris, Bristol, UK) were used. Overexpression of Activin A (*INHBA*) was achieved by retroviral transfection of cells with viral supernatant containing pBABE plasmid with zeocin resistance (Addgene, Cambridge, MA) encoding the *INHBA* gene sequence (Origene, Rockville, MD).

### Organotypic culture

Organotypic reconstructs were grown as previously described [18, 20] with the exception that each culture was rinsed in 1XPBS and incubated with Epidermalization 3 medium lacking serum for two additional days prior to harvesting. The following treatments were added to the organotypic cultures at the time of epithelial seeding and renewed with every media change: 5 ng/ml recombinant human TGFβ1, 10 ng/ml Activin A, 100 ng/ml Follistatin-288 (all from R&D Systems) or 1 μM A83-01 (Tocris).

### Scratch assays

Cells were grown to 100% confluence then a scratch was introduced using a 200 μl pipette tip. Measurement areas were marked at six different locations along the scratch. Cells were imaged at 0, 6, and 24 hours post-scratch and distance of cells traveled was measured using the Axiovision software (Carl Zeiss Microscopy, Thornwood, NY).

### Colony formation

Colony formation assays were performed by plating 500 cells in six-well plates and maintaining them in complete media for 7–8 days [61]. Cells were then fixed with 100% methanol for 10 minutes at −20°C and stained overnight in 0.1% crystal violet at room temperature. Colony counts were assessed using the GelCount™ system and software (Oxford Optronix, Abingdon, UK), courtesy of the Vanderbilt Digital Histology Shared Resource.

### Cell migration and invasion assays

Migration and Matrigel invasion chamber assays were purchased from BD Biosciences (Franklin Lakes, NJ) and performed according to manufacturer's direction. After removal of cells from the top of the membrane, cells were fixed in 100% methanol at −20°C for 10 minutes, then rinsed once in 1XPBS,. For quantification, cells were stained with 0.3% Janus green (Sigma, St. Louis, MO, cat. no. 201677) for 5 minutes at room temperature. Upon washing, cells were destained with 0.5 M HCl for 10 minutes at room temperature. The HCl solution was collected and transferred to a 96-well plate and absorbance read at 595 nm on a BioTek Synergy 4 microplate reader (BioTek Instruments, Inc., Winooski, VT). Subsequently, cells were stained in 0.1% crystal violet overnight, mounted, and imaged as previously described [18].

### Proliferation assays

Cells were plated at 1000 cells per well in a 96-well plate for proliferation assays. WST-1 reagent (Roche, Nutley, NJ) was added to each well at the time points indicated and incubated at 37°C for 1 hour. Absorbance measurements at 450 nm were taken using a BioTek Synergy 4 plate reader (BioTek Instruments, Inc.). Measurements were taken in 24-hour increments.

### ELISA

Cells were seeded at 166,000 cells per 6-well insert with full medium. The next day, cells either underwent treatment or culture media were changed to serum-free media before conditioned media was harvested 48 hours thereafter. Cell number was determined and concentration per 1 ml media was calculated and normalized per 100,000 cells. Capture ELISAs for Activin A, TGFβ1 and pan-Follistatin (FS288, FS300, FS315) were purchased from and performed following manufacturer's instructions (R&D Systems). INHA was measured using an ELISA kit purchased from Cloud Clone Corp (Houston, TX).

### Western blot

Western blots were performed as previously described [18]. Cells undergoing treatment with Activin A, Follistatin-288, A83-01, or TGFβ1 had the individual growth factors added to serum-free cell culture media for 30 minutes or 48 hours, followed by protein lysis. The results are representative of at least three independent experiments.

### Immunofluorescence

Organotypic culture tissue, previously fixed in 10% neutral buffered formalin for 24 hours and embedded in paraffin, was sectioned at 5 μm, deparaffinized, and heated in 1X TE buffer in a pressure cooker for 12 min for antigen retrieval. Samples were blocked in 1XPBS with 5% Bovine Serum Albumin (Sigma), 1XPBS-BSA, for 1 hour prior to incubation with primary antibodies in 1XPBS-BSA overnight at 4°C. Tissues were then rinsed three times in 1XPBS and incubated with secondary antibodies in 1XPBS-BSA for 1 hour at room temperature. After additional rinses with 1XPBS, the sections were mounted with Vectashield mounting medium containing DAPI (Vector Laboratories, Burlingame, CA). Images were taken on a Zeiss microscope, using Axiocam and Axiovision software (Carl Zeiss Microscopy).

Alcian Blue Staining was performed by the Translational Pathology Shared Resource at Vanderbilt University Medical Center.

### Antibodies and other reagents

SOX2, total Smad 2, phospho-Smad2, total ERK1/2 and phospho-ERK1/2 were purchased from Cell Signaling Technologies (Danvers, MA), and α-tubulin from Abcam (Cambridge, MA). Anti-TGFBR2 (clone L21) was purchased from Santa Cruz Biotechnologies (Santa Cruz, CA), E-cadherin and Keratin 8 from BD Bioscience, and vimentin from Sigma. Other antibodies used: MT1-MMP (Epitomics, Cambridge, MA) and anti-CD44 clone 2C5 (R&D Systems); SOX9 (EMD Millipore, Rockland, MA); Keratin K13 (Novus Biologicals, Littleton, CO).

### Dataset analysis

Dataset GDS1321 used to query clinical correlations with Activin A, publicly available from GEO Datasets (http://www.ncbi.nlm.nih.gov/gds/). The collected information from each dataset was analyzed and visualized in Prism version 6.00 for Mac (GraphPad software, La Jolla, California).

### Biostatical analysis

Biostatistical analysis was performed using Prism version 6.00 for Mac (GraphPad). *In vitro* and *in vivo* experiments were analyzed using Student's *t*-tests, one- or two-way ANOVAs. Statistical significance was set at *p* < 0.05. Pearson's correlation coefficients were calculated. All experiments were done in triplicates with at least three biological replicates.

## SUPPLEMENTARY FIGURES



## Abbreviations

TGFβ: transforming growth factor β
BMP: bone morphogenetic protein
EAC: esophageal adenocarcinoma
SOX: sex determining region Y box
EMT: epithelial-mesenchymal transition
INHBA: inhibin beta A subunit or Activin A
ERK: extracellular signal-regulated kinase
MAPK: mitogen activated protein kinase

## References

[1] Hyland PL, Hu N, Rotunno M, Su H, Wang C, Wang L, Pfeiffer RM, Gherman B, Giffen C, Dykes C, Dawsey SM, Abnet CC, Johnson KM, Acosta RD, Young PE, Cash BD, Taylor PR (2014). Global changes in gene expression of Barrett's esophagus compared to normal squamous esophagus and gastric cardia tissues. PLoS One.

[2] Mendelson J, Song S, Li Y, Maru DM, Mishra B, Davila M, Hofstetter WL, Mishra L (2011). Dysfunctional transforming growth factor-β signaling with constitutively active Notch signaling in Barrett's esophageal adenocarcinoma. Cancer.

[3] Onwuegbusi BA, Rees JR, Lao-Sirieix P, Fitzgerald RC (2007). Selective loss of TGFbeta Smad-dependent signalling prevents cell cycle arrest and promotes invasion in oesophageal adenocarcinoma cell lines. PLoS One.

[4] Onwuegbusi BA, Aitchison A, Chin SF, Kranjac T, Mills I, Huang Y, Lao-Sirieix P, Caldas C, Fitzgerald RC (2006). Impaired transforming growth factor beta signaling in Barrett's carcinogenesis due to frequent SMAD4 inactivation. Gut.

[5] von Rahden BH, Stein HJ, Feith M, Pühringer F, Theisen J, Siewert JR, Sarbia M (2006). Overexpression of TGF-beta1 in esophageal (Barrett's) adenocarcinoma is associated with advanced stage of disease and poor prognosis. Mol Carcinog.

[6] Milano F, van Baal JW, Buttar NS, Rygiel AM, de Kort F, DeMars CJ, Rosmolen WD, Bergman JJ, Van Marle J, Wang KK, Peppelenbosch MP, Krishnadath KK (2007). Bone morphogenetic protein 4 expressed in esophagitis induces a columnar phenotype in columnar metaplasia. Gastroenterology.

[7] van Baal JW, Milano F, Rygiel AM, Bergman JJ, Rosmolen WD, van Deventer SJ, Wang KK, Peppelenbosch MP, Krishnadath KK (2005). A comparative analysis by SAGE of gene expression profiles of Barrett's esophagus, normal squamous esophagus, and gastric cardia. Gastroenterology.

[8] Narita T, Saitoh K, Kameda T, Kuroiwa A, Mizutani M, Koike C, Iba H, Yasugi S (2000). BMPs are necessary for stomach gland formation in the chicken embryo: a study using virally induced BMP-2 and Noggin expression. Development.

[9] Que J, Choi M, Ziel JW, Klingensmith J, Hogan BL (2006). Morphogenesis of the trachea and esophagus: current players and new roles for noggin and Bmps. Differentiation.

[10] Litingtung Y, Lei L, Westphal H, Chiang C (1998). Sonic hedgehog is essential to foregut development. Nat. Genet.

[11] Castillo D, Puig S, Iglesias M, Seoane A, de Bolós C, Munitiz V, Parrilla P, Comerma L, Poulsom R, Krishnadath KK, Grande L, Pera M (2012). Activation of the BMP4 pathway and early expression of CDX2 characterize non-specialized columnar metaplasia in a human model of Barrett's esophagus. J Gastrointest Surg.

[12] Shaker A, Binkley J, Darwech I, Swietlicki E, McDonald K, Newberry R, Rubin DC (2013). Stromal cells participate in the murine esophageal mucosal injury response. Am J Physiol Gastrointest Liver Physiol.

[13] Isohata N, Aoyagi K, Mabuchi T, Daiko H, Fukaya M, Ohta H, Ogawa K, Yoshida T, Sasaki H (2009). Hedgehog and epithelial-mesenchymal transition signaling in normal and malignant epithelial cells of the esophagus. Int J Cancer.

[14] Jethwa P, Naqvi M, Hardy RG, Hotchin NA, Roberts S, Spychal R, Tselepis C (2008). Overexpression of Slug is associated with malignant progression of esophageal adenocarcinoma. World J Gastroenterol.

[15] Tomizawa Y, Wu TT, Wang KK (2012). Epithelial mesenchymal transition and cancer stem cells in esophageal adenocarcinoma originating from Barrett's esophagus. Oncol Lett.

[16] Rees JR, Onwuegbusi BA, Save VE, Alderson D, Fitzgerald RC (2006). *In vivo* and *in vitro* evidence for transforming growth factor-beta1-mediated epithelial to mesenchymal transition in esophageal adenocarcinoma. Cancer Res.

[17] Seder CW, Hartojo W, Lin L, Silvers AL, Wang Z, Thomas DG, Giordano TJ, Chen G, Chang AC, Orringer MB, Beer DG (2009). INHBA overexpression promotes cell proliferation and may be epigenetically regulated in esophageal adenocarcinoma. J Thorac Oncol.

[18] Le Bras GF, Loomans HA, Taylor CJ, Revetta FL, Andl CD (2014). Activin A balance regulates epithelial invasiveness and tumorigenesis. Lab Invest.

[19] Klingbeil P, Isacke CM (2011). The “alternative” EMT switch. Breast Cancer Research: BCR.

[20] Le Bras GF, Allison GL, Richards NF, Ansari SS, Washington MK, Andl CD (2011). CD44 upregulation in E-cadherin-negative esophageal cancers results in cell invasion. PLOS ONE.

[21] Amthor H, Nicholas G, McKinnell I, Kemp CF, Sharma M, Kambadur R (2004). Follistatin complexes Myostatin and antagonizes Myostatin-mediated inhibition of myogenesis. Developmental Biology.

[22] Iemura S, Yamamoto TS, Takagi C, Uchiyama H, Natsume T, Shimasaki S (1998). Direct binding of follistatin to a complex of bone-morphogenetic protein and its receptor inhibits ventral and epidermal cell fates in early Xenopus embryo. Proceedings of the National Academy of Sciences of the United States of America.

[23] Nogai H, Rosowski M, Grun J, Rietz A, Debus N, Schmidt G (2007). Follistatin antagonizes transforming growth factor-β3-induced epithelial-mesenchymal transition *in vitro*: implications for murine palatal development supported by microarray analysis. Differentiation.

[24] Lord RV, Wickramasinghe K, Long TI, Kurumboor SK, Bernstein L, Peters JH, DeMeester SR, DeMeester TR, Skinner KA, Laird PW, Eads CA1, (2001). Epigenetic patterns in the progression of esophageal adenocarcinoma. Cancer Res.

[25] Boch JA, Shields HM, Antonioli DA, Zwas F, Sawhney RA, Trier JS (1997). Distribution of cytokeratin markers in Barrett's specialized columnar epithelium. Gastroenterology.

[26] Clemons NJ, Wang DH, Croagh D, Tikoo A, Fennell CM, Murone C, Scott AM, Watkins DN, Phillips WA (2012). Sox9 drives columnar differentiation of esophageal squamous epithelium: a possible role in the pathogenesis of Barrett's esophagus. Am J Physiol Gastrointest Liver Physiol.

[27] Song S, Maru DM, Ajani JA, Chan CH, Honjo S, Lin HK, Correa A, Hofstetter WL, Davila M, Stroehlein J, Mishra L (2013). Loss of TGFβ and β2SP activates notch signaling and SOX9 expression in esophageal adenocarcinoma. Cancer Res.

[28] Eloubeidi MA, Mason AC, Desmond RA, El-Serag HB (2003). Temporal trends (1973–1997) in survival of patients with esophageal adenocarcinoma in the United States: a glimmer of hope?. Am. J. Gastroenterol.

[29] Li X (2014). Temporal and Spatial evolution of somatic chromosomal alterations: a case-cohort study of Barrett's esophagus. Cancer Prev. Res. (Phila).

[30] Weaver JM (2014). Ordering of mutations in preinvasive disease stages of esophageal carcinogenesis. Nat. Genet.

[31] Ramos P, Bentires AM (2015). Mechanism-based cancer therapy: resistance to therapy, therapy for resistance. Oncogene.

[32] Nones K (2014). Genomic catastrophes frequently arise in esophageal adenocarcinoma and drive tumorigenesis. Nature Communications.

[33] Agrawal N (2012). Comparative genomic analysis of esophageal adenocarcinoma and squamous cell carcinoma. Cancer Discovery.

[34] Ryu B, Kern SE (2003). The essential similarity of TGFbeta and activin receptor transcriptional responses in cancer cells. Cancer Biol Ther.

[35] Matzuk MM, Kumar TR, Vassalli A, Bickenbach JR, Roop DR, Jaenisch R, Bradley A (1995). Functional analysis of activins during mammalian development. Nature.

[36] Matzuk MM, Kumar TR, Bradley A (1995). Different phenotypes for mice deficient in either activins or activin receptor type II. Nature.

[37] Pawlowski SA, Wiles MV, Yin M, Boivin GP, Howles PN, Ding J, Ferguson MW, Doetschman T, Proetzel G 1995 Nat genetics Proetzel G1 (1995). Transforming growth factor-beta 3 is required for secondary palate fusion. Nat Genet.

[38] Sanford LP, Ormsby I, Gittenberger-de Groot AC, Sariola H, Friedman R, Boivin GP, Cardell EL, Doetschman T (1997). TGFbeta2 knockout mice have multiple developmental defects that are non-overlapping with other TGFbeta knockout phenotypes. Development.

[39] Hempen PM, Zhang L, Bansal RK, Iacobuzio-Donahue CA, Murphy KM, Maitra A, Vogelstein B, Whitehead RH, Markowitz SD, Willson JK, Yeo CJ, Hruban RH, Kern SE (2003). Evidence of selection for clones having genetic inactivation of the activin A type II receptor (ACVR2) gene in gastrointestinal cancers. Cancer Res.

[40] Su GH, Bansal R, Murphy KM, Montgomery E, Yeo CJ, Hruban RH, Kern SE (2001). ACVR1B (ALK4, activin receptor type 1B) gene mutations in pancreatic carcinoma. Proc Natl Acad Sci U S A.

[41] Kodach LL, Wiercinska E, de Miranda NF, Bleuming SA, Musler AR, Peppelenbosch MP, Dekker E, van den Brink GR, van Noesel CJ, Morreau H, Hommes DW, Ten Dijke P, Offerhaus GJ, Hardwick JC (2008). The bone morphogenetic protein pathway is inactivated in the majority of sporadic colorectal cancers. Gastroenterology.

[42] Sekiya T, Adachi S, Kohu K, Yamada T, Higuchi O, Furukawa Y, Nakamura Y, Nakamura T, Tashiro K, Kuhara S, Ohwada S, Akiyama T (2004). Identification of BMP and activin membrane-bound inhibitor (BAMBI), an inhibitor of transforming growth factor-beta signaling, as a target of the beta-catenin pathway in colorectal tumor cells. J Biol Chem.

[43] Fritzmann J, Morkel M, Besser D, Budczies J, Kosel F, Brembeck FH, Stein U, Fichtner I, Schlag PM, Birchmeier W (2009). A colorectal cancer expression profile that includes transforming growth factor beta inhibitor BAMBI predicts metastatic potential. Gastroenterology.

[44] Boonstra JJ, van Marion R, Douben HJ, Lanchbury JS, Timms KM, Abkevich V, Tilanus HW, de Klein A, Dinjens WN (2012). Mapping of homozygous deletions in verified esophageal adenocarcinoma cell lines and xenografts. Genes Chromsom Cancer.

[45] Dulak AM, Stojanov P, Peng S, Lawrence MS, Fox C, Stewart C, Bandla S, Imamura Y, Schumacher SE, Shefler E, McKenna A, Carter SL, Cibulskis K, Sivachenko A, Saksena G, Voet D, Ramos AH, Auclair D, Thompson K, Sougnez C, Onofrio RC, Guiducci C, Beroukhim R, Zhou Z, Lin L, Lin J, Reddy R, Chang A, Landrenau R, Pennathur A, Ogino S, Luketich JD, Golub TR, Gabriel SB, Lander ES, Beer DG, Godfrey TE, Getz G, Bass AJ (2013). Exome and whole-genome sequencing of the esophagus, stomach, and colon exhibit distinct patterns of genome instability and oncogenesis. Nature Genetics.

[46] Rejon CA, Hancock MA, Yining NL, Thompson TB, Hebert TE, Bernard DJ (2013). Acitvins bind and signal via bone morphogenic protein receptor type II (BMPR2) in immortalized gonadotrope-like cells. Cell Sig.

[47] Goumans MJ, Valdimarsdottir G, Itoh S, Rosendahl A, Sidera P, ten Dijke P (2002). Balancing the activation state of the endothelium via two distinct TGF-beta type I receptors. EMBO J.

[48] Yoshinaga K, Yamashita K, Mimori K, Tanaka F, Inoue H, Mori M (2008). Activin a causes cancer cell aggressiveness in esophageal squamous cell carcinoma cells. Ann Surg Oncol.

[49] Yoshinaga K, Mimori K, Inoue H, Kamohara Y, Yamashita K, Tanaka F, Mori M (2008). Activin A enhances MMP-7 activity via the transcription factor AP-1 in an esophageal squamous cell carcinoma cell line. Int J Oncol.

[50] Saadi A, Shannon NB, Lao-Sirieix P, O'Donovan M, Walker E, Clemons NJ, Hardwick JS, Zhang C, Das M, Save V, Novelli M, Balkwill F, Fitzgerald RC (2010). Stromal genes discriminate preinvasive from invasive disease, predict outcome, and highlight inflammatory pathways in digestive cancers. Proc Natl Acad Sci U S A.

[51] Lao-Sirieix P, Fitzgerald RC (2010). Role of the microenvironment in Barrett's carcinogenesis. Biochem Soc Trans.

[52] Wang DH, Tiwari A, Kim ME, Clemons NJ, Regmi NL, Hodges WA, Berman DM, Montgomery EA, Watkins DN, Zhang X, Zhang Q, Jie C, Spechler SJ, Souza RF (2014). Hedgehog signaling regulates FOXA2 in esophageal embryogenesis and Barrett's metaplasia. J Clin Investigation.

[53] Xiao L, Yuan X, Sharkis SJ (2006). Activin A maintains self-renewal and regulates fibroblast growth factor, Wnt, and bone morphogenic protein pathways in human embryonic stem cells. Stem Cells.

[54] Kowanetz M, Valcourt U, Bergström R, Heldin CH, Moustakas A (2004). Id2 and Id3 define the potency of cell proliferation and differentiation responses to transforming growth factor beta and bone morphogenetic protein. Mol Cell Biol.

[55] Wang DH, Clemons NJ, Miyashita T, Dupuy AJ, Zhang W, Szczepny A, Corcoran-Schwartz IM, Wilburn DL, Montgomery EA, Wang JS, Jenkins NA, Copeland NA, Harmon JW, Phillips WA, Watkins DN (2010). Aberrant epithelial-mesenchymal Hedgehog signaling characterizes Barrett's metaplasia. Gastroenterology.

[56] Pham TH, Genta RM, Spechler SJ, Souza RF, Wang DH (2014). Development and characterization of a surgical mouse model of reflux esophagitis and Barrett's esophagus. J Gastrointest Surg.

[57] Song S, Ajani JA, Honjo S, Maru DM, Chen Q, Scott AW, Heallen TR, Xiao L, Hofstetter WL, Weston B, Lee JH, Wadhwa R, Sudo K, Stroehlein JR, Martin JF, Hung MC, Johnson RL (2014). Hippo coactivator YAP1 upregulates SOX9 and endows esophageal cancer cells with stem-cell like properties. Cancer Res.

[58] Zhao Y, Bao Q, Schwarz B, Zhao L, Mysliwietz J, Ellwart J, Renner A, Hirner H, Niess H, Camaj P, Angele M, Gros S, Izbicki J, Jauch KW, Nelson PJ, Bruns CJ (2014). Stem cell-like side populations in esophageal cancer: a source of chemotherapy resistance and metastases. Stem Cells Dev.

[59] Kim AH, Lebman DA, Dietz CM, Snyder SR, Eley KW, Chung TD (2003). Transforming growth factor-beta is an endogenous radioresistance factor in the esophageal adenocarcinoma cell line OE-33. Int J Oncol.

[60] Hughes SJ, Nambu Y, Soldes OS, Hamstra D, Rehemtulla A, Iannettoni MD, Orringer MB, Beer DG (1997). Fas/APO-1 (CD95) is not translocated to the cell membrane in esophageal adenocarcinoma. Cancer Res.

[61] Franken NAP, Rodermond HM, Stap J, Haveman H, van Bree C (2006). Clonogenic assays of cells *in vitro*. Nature Protocols.

