# Supplementary material for: Activin a signaling regulates cell invasion and proliferation in esophageal adenocarcinoma
Source: Oncotarget. 2015 Oct 6;6(33):34228–44. doi: 10.18632/oncotarget.5349 (PMC4741448; doi:10.18632/oncotarget.5349)
Supplement: Supplementary file 1 [file oncotarget-06-34228-s001.pdf]

SUPPLEMENTARY FIGURES

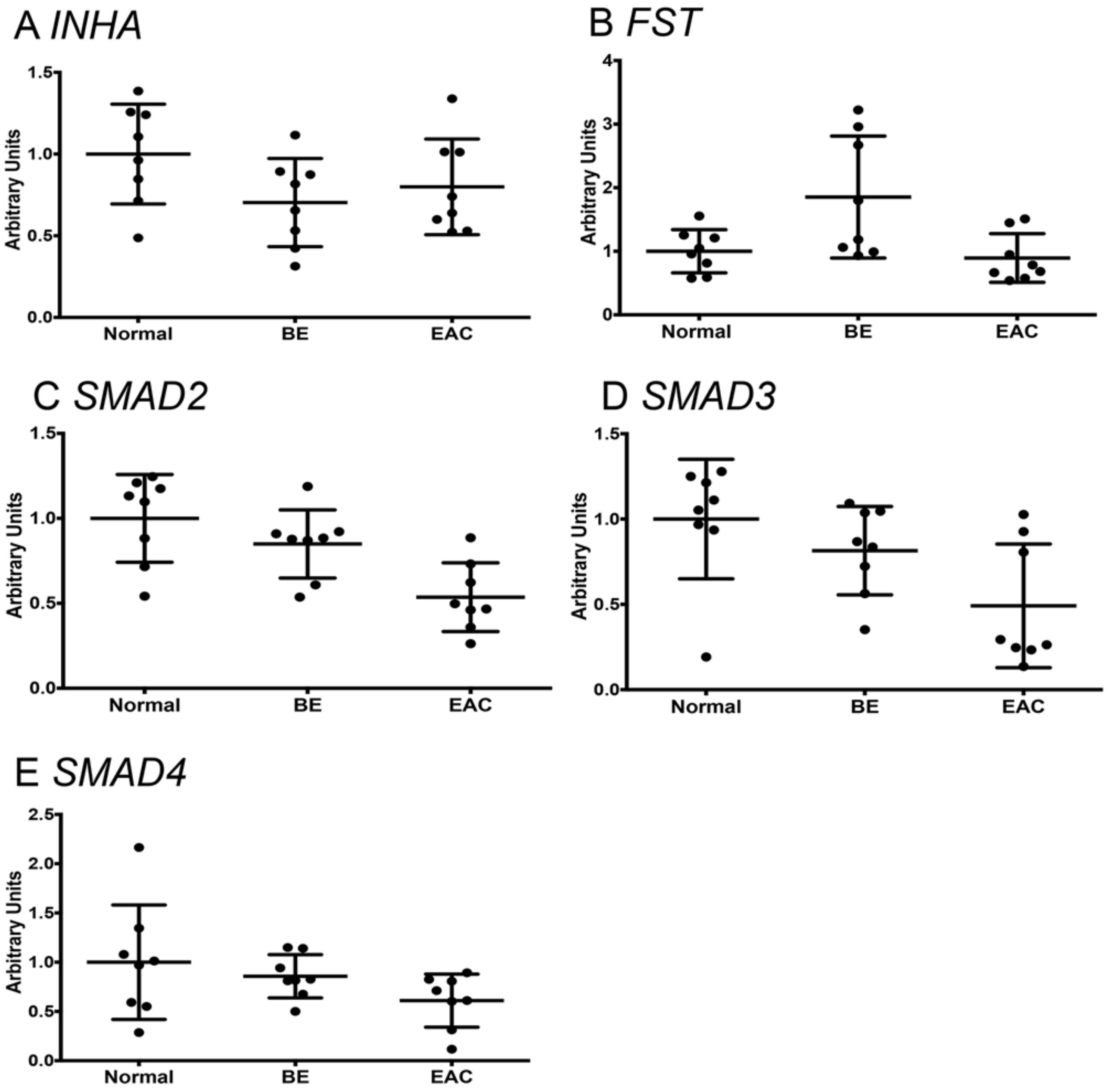

Supplementary Figure S1: Analysis of expression levels of components of the Activin A signaling pathways in the progression from normal esophagus to Barrett's esophagus and esophageal adenocarcinoma. Comparison of the expression for *INHA* (the inhibin α subunit that forms heterodimers with inhibin β_A, resulting in the Inhibin A ligand, an Activin A inhibitor), the antagonist *FST* and downstream canonical signaling targets *SMAD4*, *SMAD2* and *SMAD3* based a publicly available GEO dataset (accession number GDS1321). Values were measured from extracted and purified RNA, shown here as arbitrary units. Pearson's correlation coefficients were calculated: *INHA* $y = -0.1002x+1.0347$, r2=0.43832; FST $y = -0.0513x +1.3548$, r2=0.01021; *SMAD4* $y = -0.195x+1.2125$, r2=0.97645; *SMAD2* $y = -0.232x+1.2591$, r2=0.96066; *SMAD3* $y = -0.2543x+1.2773$, r2=0.97617.

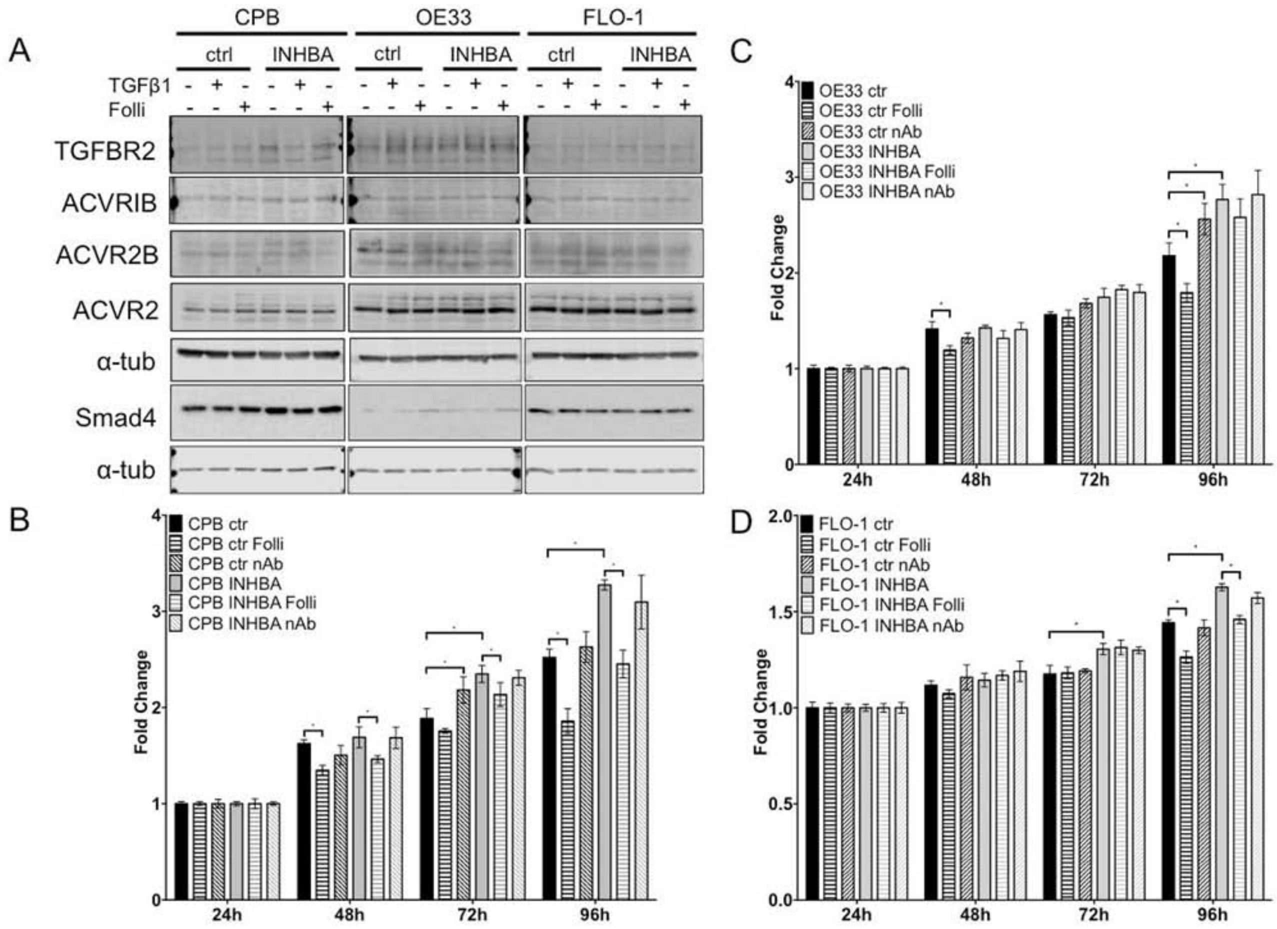

Supplementary Figure S2: Protein expression of receptor complex components is unchanged upon *INHBA* overexpression, yet proliferation is altered. A. Protein lysates were collected after 48 hours after treatment with TGFβ1 or Follistatin and used for Western blot. Analysis of components of the Activin A and TGFβ receptor complexes (TGFBR2, ACVR1B, AVCR2 AVCR2B) showed no changes in expression following *INHBA* overexpression. Smad4 expression is lower in OE33 cells than CPB and FLO-1 cells. TGFBR2, ACVRIB, AVCR2B were probed on the same membrane as α-tubulin for Smad4 (bottom panel). Cell viability was measured by WST-1 assay over the course of 96 hours. **B.** CPB cells with *INHBA* overexpression have higher proliferation rates, which are reduced in the presence of Follistatin, but not an Activin A neutralizing antibody (nAb). **C.** OE33 cells and OE33-*INHBA* cells respond to Follistatin with reduced proliferation rates, but not nAb. Overall, INHBA overexpression increases cell growth. **D.** FLO-1 cells with INHBA overexpression have higher proliferation rates, which are reduced in the presence of Follistatin, but not Act A neutralizing antibody. Data from three independent replicates were pooled and two-way ANOVA performed. All proliferation treatment conditions were normalized to their respective 24-hour time-point and the fold change was calculated from this value. $*p < 0.05$.

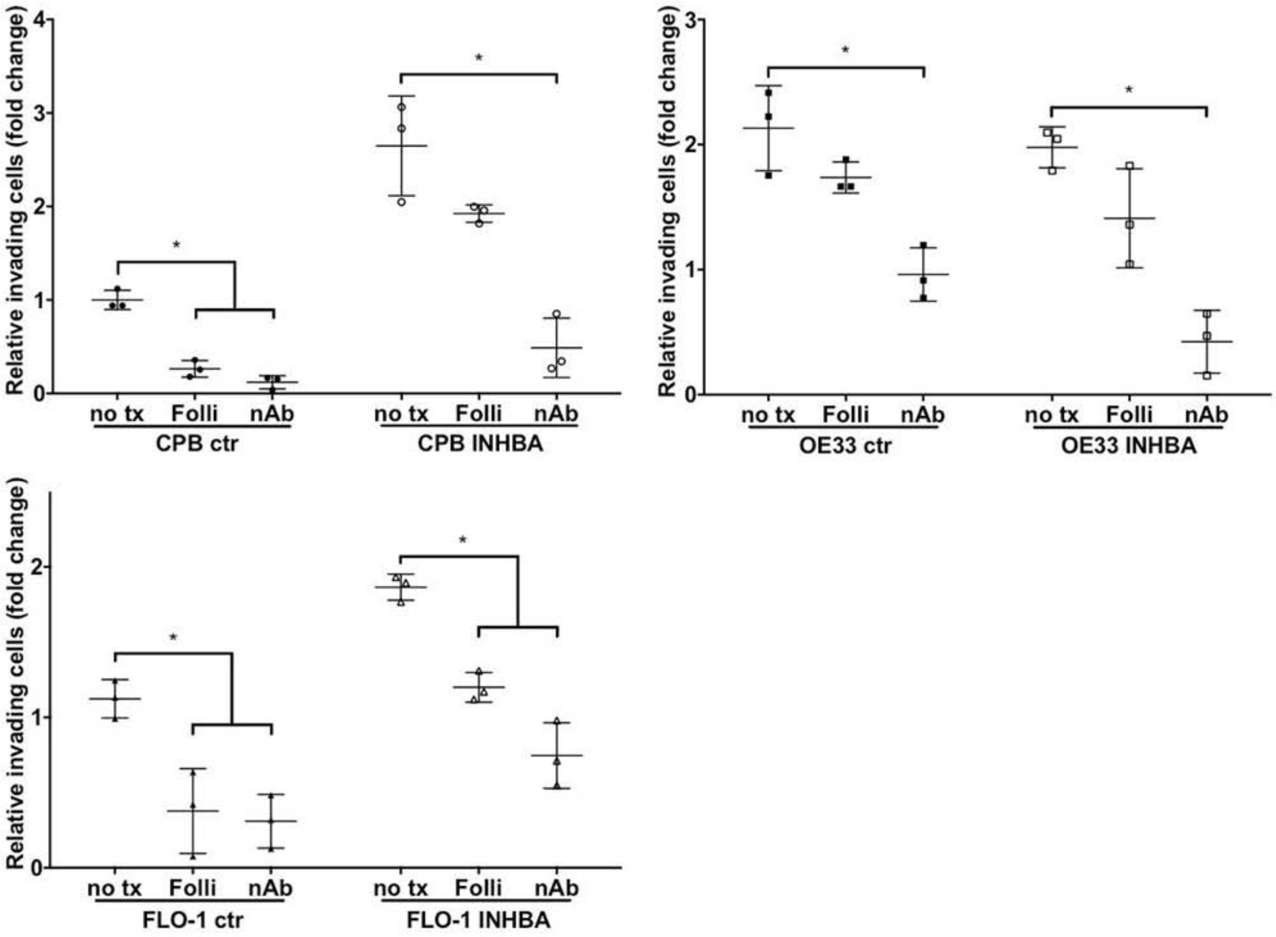

Supplementary Figure S3: Cell invasion of control and INHBA overexpressing cells is inhibited by Follistatin and neutralizing antibody against Activin A. Empty vector control (ctr) and INHBA overexpressing cells were grown in Boyden chamber invasion assays in the presence or absence of Follistatin (Folli) or Activin A neutralizing antibody (nAb) and compared to untreated controls (no tx). Both treatments reduced the invasive capabilities of the cells, yet nAb is more effective than Follistatin. Statistical analysis was performed using Student's *t*-test, $*p < 0.05$.

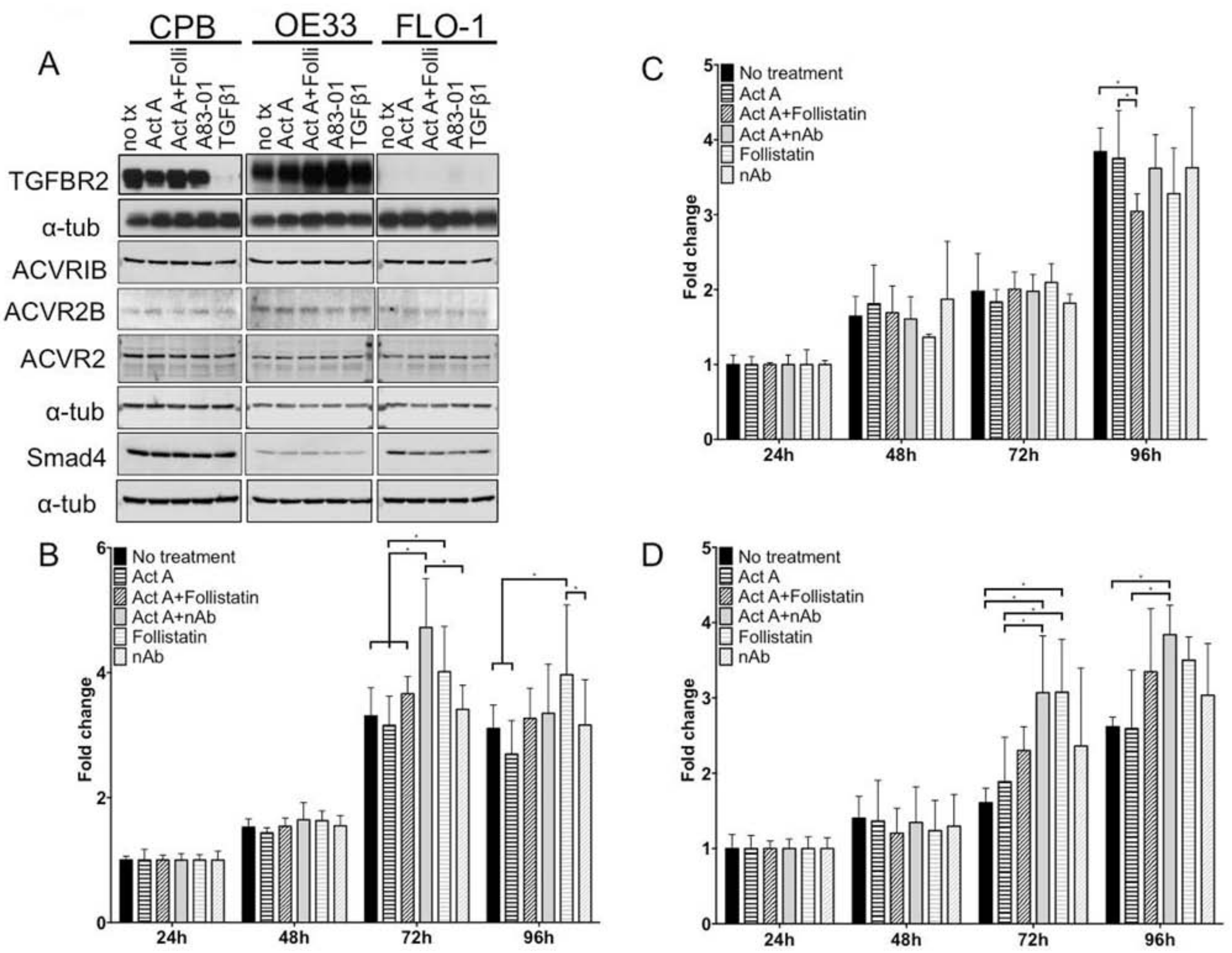

Supplementary Figure S4: Protein expression level of receptor complex components are unchanged upon Activin A treatment, however proliferation is altered. A. Protein lysates were collected after 48 hours and antibodies against TGFBR2, AVCR1B, ACVR2, ACVR2B, and Smad4 used to determine if changes in protein expression occur following stimulation with Activin A, TGFβ (as a positive control), Follistatin (Folli), or A83-01. No changes were noted aside from TGFBR2, which was downregulated in CPB cells upon TGFβ stimulation. Membrane for AVCR1B is the same as for Smad4. α-Tubulin on the bottom panel serves as ACVR1B loading control. Cell viability was measured by WST-1 assay over the course of 96 hours. Cells were treated with either Activin A (Act A), the combination of Act A and Follistatin, Act A and Activin A neutralizing antibody (nAb) or each alone. **B.** CPB cells, in response to Follistatin showed increased cell proliferation. **C.** OE33 cell proliferation was decreased in response to the combination of Act A and Follistatin. **D.** FLO-1 cells in response to Activin A stimulation showed increased cell proliferation in the presence of Activin A and Folistatin or the combination of Act A and neutralizing antidboy (nAb). Data from three experiments were pooled and a two-way ANOVA was performed. All proliferation treatment conditions were normalized to their respective 24-hour time point and the fold change was calculated from this value. *$p < 0.05$.

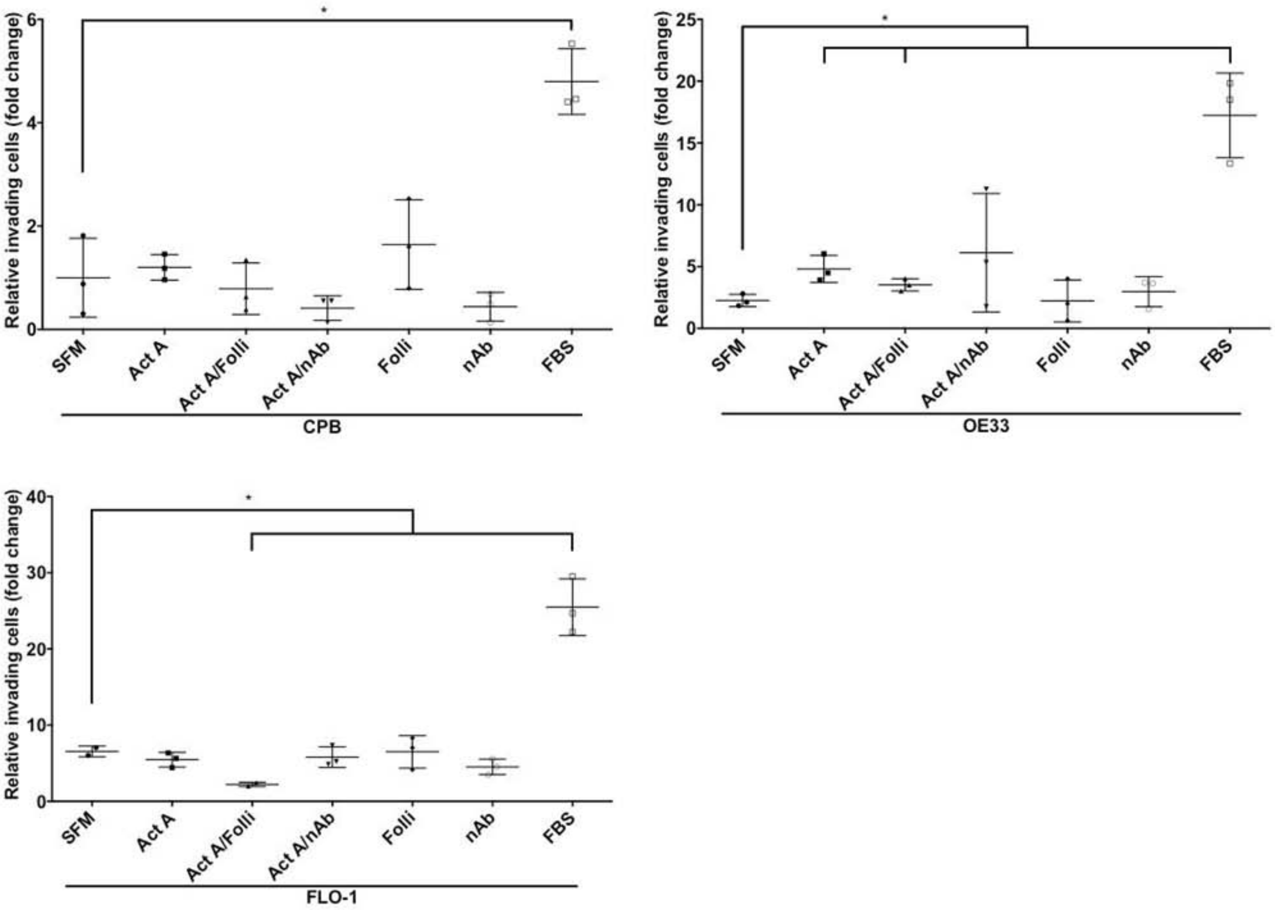

Supplementary Figure S5: Cell invasion is inhibited when adding Activin A in combination with Follistatin in FLO-1 cells. Cells were grown in serum-free media in Boyden chamber invasion assays with either Activin A (ActA), the combination of Activin A and Follistatin, Activin A and an Activin A neutralizing antibody (nAb) or each alone in the bottom chamber. FBS was used as a positive control. The invasive potential of OE33 cells was increased by the combination of ActA and Follistatin. In FLO-1 cells, ActA and Follistatin reduced the invasive potential, while FBS increased it in all cell lines. Post-hoc statistical analysis comparing all the conditions was performed using one-way ANOVA. *$p < 0.05$.
